# Radiofrequency Treatment of Labia Minora and Majora: A Minimally Invasive Approach to Vulva Restoration

**DOI:** 10.1097/GOX.0000000000002418

**Published:** 2020-04-22

**Authors:** Erez Dayan, Henry Ramirez, Spero Theodorou

**Affiliations:** * Massachusetts General Hospital, Division of Plastic and Reconstructive Surgery, Harvard Medical School, Boston, Mass.; † Southern Oklahoma Women’s Heath, Private Practice, Ardmore, Okla.; ‡ Manhattan, Eye, Ear, and Throat Hospital, Division of Plastic and Reconstructive Surgery, New York, N.Y.

## Abstract

Labiaplasty has rapidly increased in popularity over the past 5 years.Traditional labiaplasty is associated with potential complications, such as dehiscence, hematoma, flap necrosis, narrowed introitus, pain, and asymmetry. Minimally invasive techniques such as radiofrequency (RF) have emerged as viable alternatives to traditional labiaplasty through a temperature-controlled bipolar mechanism to heat tissues to target temperatures of 40°C–45°C. This controlled energy delivery leads to an inflammatory cascade initiating neocollagenesis, angiogenesis, and elastogenesis over the coming 3–4 months. A single surgeon series of labia minora and majora treatment by RF (InMode, Lake Forrest, Calif.) was reviewed between April 2018–October 2018. Demographic data were collected as well grade of hypertrophy (pre/posttreatment), number of vaginal deliveries, and reason for treatment. Procedural parameters were recorded, including internal/external temperatures, total energy used, and time of treatment. All adverse events were recorded. Objective and subjective data points were obtained in the form of patient surveys and photographic evaluation by lay persons as well as plastic surgeons objective to the treatment. Ten consecutive patients were treated with bipolar RF (InMode, Lake Forrest, Calif.) between April 2018–October 2018. Mean age was 44 (29–54). Average number of pregnancies was 2 (STD 1.1). Three patients were treated for aesthetic concerns, 3 for functional complaints, and 4 desired improvement in both. Overall graded improvement in labia size/contour was +50% (STD ±15.3). Patient satisfaction scale data demonstrated 9.5/10 (±1.7). All patients (10/10) stated that they would undergo treatment again. In all cases, the surgeon observed tightening of the clitoral hood, introitus, forchett, as well as improved distribution of dark pigmentation of the labia minora. There were no significant complications and no need for additional procedures. Average recovery time was 14 days (STD 2.2). Treatment of labia hyperplasia and laxity with bipolar RF may potentially fill a treatment gap of women seeking aesthetic and functional improvements without surgical labiaplasty. A powered prospective randomized double-blinded study is needed to further elucidate the role of this technology.

## INTRODUCTION

Labiaplasty has rapidly increased in popularity for the management of functional and aesthetic problems associated with prominent labia.^1^ The first description in the plastic surgery literature was by Hodgkinson and Hait (1984) who identified that labia minora protrusion past labia majora is aesthetically and functionally unsatisfactory.^1^ According to the American Society of Plastic Surgeons, 12,000 labiaplasty were performed in 2016, up 40% from the prior year.^2^ Demographic data show trends of younger women (<18 years) seeking labiaplasty.^3^ This growth has been attributed to decreased stigmatization, changes in fashion trends, and increased exposure to nudity in social media.^4^

Studies show that women seek labiaplasty for a variety of reasons; including aesthetic purposes (1/3), functional impairment (ie, pain, discomfort, difficulty maintaining hygiene) (1/3), and a combination of both (1/3).^1,4^ Patient satisfaction rates in the literature are consistently 90%–95%.^4^ However, shortcomings have included the lack of measurable standards of care, paucity of evidence based outcomes, and inconsistent terminology.^5^ Many surgical techniques have been described in the literature to treat labia hypertrophy (ie, deepithelialization, direct excision, Z-plasty, etc).^1,3–7^ All operations carry their inherent advantages/disadvantages, and there is little evidence to guide which is best for a given deformity. Potential complications of surgical labiaplasty include scarring, irregular edges, over-resection, wound dehiscence, narrowed introitus, pain, paresthesia, dryness, and asymmetry.^1,5,7^

Nonsurgical options for vulvovaginal rejuvenation are among the fastest growing areas in plastic surgery.^6^ A number of energy-based devices, including radiofrequency (RF) and laser (CO_2_, Er:YAG) have been used to improve external genital appearance, vaginal laxity, and pelvic floor dysfunction (ie, urinary incontinence).^8^ Patients and clinicians often view these minimally invasive options as more attractive to standard operative treatment. RF applied to the vulvovaginal tissue has been shown to stimulate proliferation of glycogen enriched epithelium, neovascularization, and collagen formation by creating heat via impedance, as an electric current is conducted through the target tissues.^6^ Once these devices generate temperatures between 38°C and 42°C, an inflammatory cascade is initiated to induce these changes over the subsequent 3–4 months.^1,4^ The purpose of this article is to describe our experience with bipolar RF for the treatment of prominent labia minora and majora, with a focus on efficacy and safety.

## METHODS

A single surgeon series of labia minora and majora treatment by RF (Facetite modified to Accutite, InMode, Lake Forest, Calif.) was reviewed between April 2018 and October 2018. Demographic data were collected as well as degree of labia hypertrophy, protrusion, number of vaginal deliveries, history of trauma (ie, episiotomy), and reason for treatment. Procedural parameters were recorded, including internal/external temperatures, total energy used, time of treatment, and perioperative medications used. All adverse events were documented. Results were assessed using objective and subjective data points including a patient satisfaction survey and photographic evaluation by 2 independent plastic surgeons impartial to the treatment.

## TREATMENT PROTOCOL

A detailed medical history and physical was obtained on all patients before treatment. Patients in our series were classified into one of three “treatment gap” groups: (1) women who do not want traditional invasive surgery, (2) women who had prior surgery but suffer from recurrent laxity, and (3) women with modest labia hypertrophy but not severe enough to justify traditional excision. Exclusion criteria included: unrealistic expectations, open wounds, active infection, dermatologic conditions, bleeding disorders, collagen disorders, history of keloids/hypertrophic scaring, and immunocompromised state. The distance from midline to free edge of the labia minora when extended laterally was measured to assess pre and posttreatment labia hypertrophy. While there has not been consensus on this measurement, early definition of labia minora hypertrophy included a distance of >5cm.^9^ More recently, it has been proposed that this distance should be reduced to 3 or 4 cm.^10^ We recorded this measurement of labia laxity as an objective data point, but candidacy for RF labiaplasty was primarily based on symptomatology. Additionally, we measured labia protrusion as described by Motakef et al based on the distance of the lateral edge of the labia minora from that of the labia majora rather than the introitus. This scale categorizes labial protrusion as class I (0–2 cm), class II (2–4 cm), and class III (>4 cm).^11^

All patients were premedicated with 10 mg of oral diazepam and 5/325mg of hydrocodone with acetaminophen. One dose of oral antibiotics was given preoperatively (cephalexin or ciprofloxacin). The patient was positioned in either a frog-leg position or in stirrups. After standard prep and draping, access points were injected at the caudal aspect of each labia (majora and minora) with 2–5 ml of local anesthesia (1% lidocaine with 1:200,000 epinephrine). Next a 14-gauge needle was used to create puncture site access. A 20-gauge spinal needle was used to infiltrate 20–40 ml of tumescent solution per treatment site (50 ml of 2% lidocaine, 12 ml sodium bicarbonate, 1.5 mg epinephrine per liter of lactate ringers). Hydrosoluble lubricating gel was placed over the labia to improve transduction and gliding between the 2 ports of the RF device. The RF settings included a controlled internal temperature cutoff at 60°C and 37°C externally. The 40 W bipolar RF cannula (Facetite modified to Accutite, InMode, Lake Forest, Calif.) was placed into the access port and moved in a radial cranio-caudal motion until the tissues reached target temperature. (See Video 1, [online], which displays bipolar RF treatment of labia.) (See Video 2, [online], which displays an animation of the bipolar RF treatment of labia.) This was done to systematically treat segments across the labia. Treatment was stopped ~1.5 cm from the access port to avoid repeat heating. Audible and visual cues from the device were used to guide treatment (faster beeping indicate that the clinician is reaching target temperatures). Once target temperature was reached, treatment continued for 1 minute and then was stopped.

**Video 1.** Video 1 from "Radiofrequency treatment of Labia Minora and Majora: a minimally invasive approach to vulva restoration"

**Video 2.** Video 2 from "Radiofrequency treatment of Labia Minora and Majora: a minimally invasive approach to vulva restoration"

## RESULTS

**Fig. 1.**
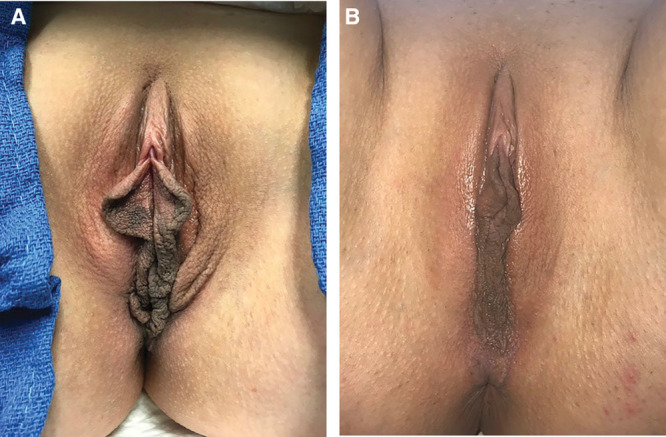
Preoperative presentation (A) and 8 month postoperative (B) result after bipolar RF labiaplasty.

Ten consecutive patients were treated with bipolar RF (Facetite modified to Accutite, InMode, Lake Forest, Calif.) between April 2018 and October 2018. Mean age was 44 (29–54). Average number of pregnancies was 2 (STD 1.1). Three patients were treated for aesthetic concerns, 3 for functional complaints, and 4 desired improvement in both. (Table 1) Mean follow-up time was 8 months (±2.1 months). Preoperative measurements of labia hypertrophy and protrusion had mean of 4.4 cm (±1.3) and 3.9 (±2.3), respectively. Measurements were obtained for all patients 6 months post procedure with a measured average improvement of 2.7 (±2.2) and 3.1 (±2.3) representing a +38.6% (STD ±15.3) and 20.5% (STD ±17.4) change. A patient satisfaction scale [1 (unsatisfied)–10 (most satisfied)] demonstrated a score of 9.5/10 (±1.7), indicating that most patients were highly satisfied with the procedure outcome. All patients (10/10) stated that they would undergo treatment again. In all cases, the surgeon observed tightening of the clitoral hood, introitus, forchett, as well as improved distribution of dark pigmentation of the labia minora (Fig. 1). There were no significant complications and no need for additional procedures. Average recovery time was 14 days (STD 2.2).

**Table 1.** Demographic Data and Pre-postoperative Measurements on Bipolar RF Labiaplasty Patients

Age	
Labia hypertrophy	4.4 cm (±1.3)
Labia protrusion	3.9 (±2.3)
Vaginal deliveries	2 (±1.7)
History of trauma	50%
Reason for treatment	Aesthetic 30%
	Functional 30%
	Both
	40%

## DISCUSSION

RF treatment for skin laxity was first studied nearly 70 years ago.^6^ However, application to the external female genitalia has only emerged in the last decade.^6^ The use of RF in this region is of particular interest as the contractile effect is known to increase in naturally moist tissues.^1,8^ Despite traditionally high satisfaction rates with surgical labiaplasty, there are associated risks and downtime. RF avoids complications of traditional labiaplasty, including unfavorable scarring, flap necrosis, hematoma, and over-resection.^4^ However, given the generation of heat, there is a risk of burns with RF not seen with traditional labiaplasty. As seen in our study, return to normal sexual function and activities was 14 days compared with the 30–45 days typically cited after traditional labiaplasty.^5^

Using the temperature controlled device at 40°C–45°C, collagen denatures and regenerates over 3–4 months to provide an increased amount and strength of collagen/elastin, leading to long-term tightening. There is an immediate tissue tightening observed with RF treatment. This is explained by collagen contraction, causing the triple helix structure to fold, creating shorter and thicker collagen fibers.^6,8^ Consistent with our study’s findings, Lordella et al used bipolar RF on women with labial laxity; all patients reported satisfaction with treatment outcome in regards to sexual function, lubrication, and arousal.^6–8^ Vanaman et al confirmed these changes histologically in a vulvovaginal tissue after treatment with RF.^12^ Ovid models have shown the same findings.^6–8^

**Fig. 2.**
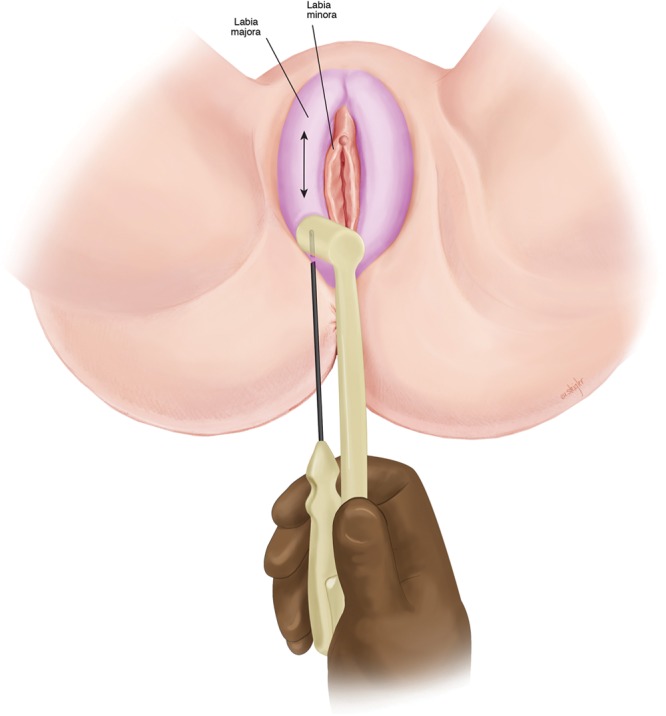
Treatment of labia majora with bipolar RF.

**Fig. 3.**
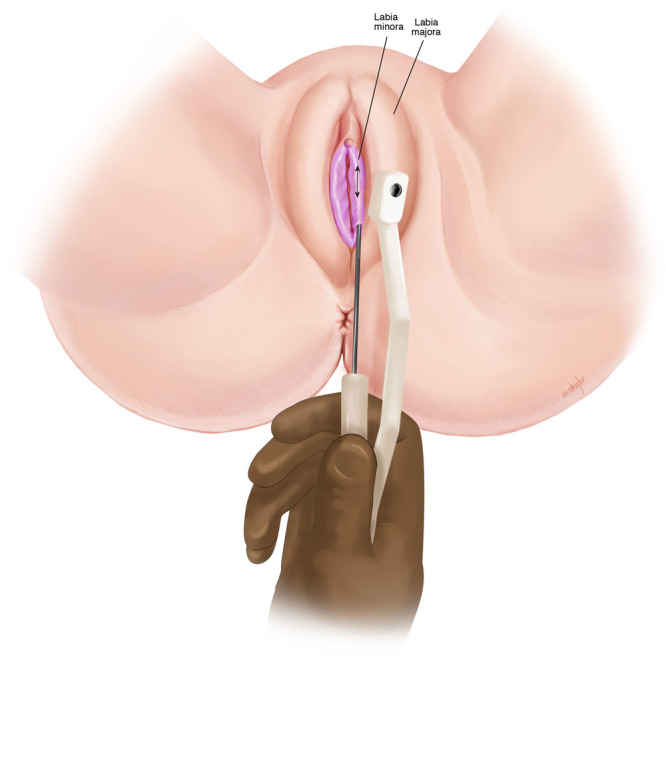
Treatment of labia minora with bipolar RF.

Uniquely, in our cohort of patients, the labia majora (Fig. 2) were treated in addition to the labia minora (Fig. 3), as there is often soft tissue laxity in this region as well. In traditional labiaplasty, there are limited surgical options for treatment of labia majora. We hypothesize that treatment of labia majora in addition to minora allows for a “Boa” contractile effect—analogous to the gradual action of a boa constrictor. This theory explains our observation of tighter clitoral hood, introitus, and forchette.

Our study showed no significant complications and an overall 50% improvement in labia hypertrophy and laxity with >95% patient satisfaction. This is in line with most clinical trials that exist using energy based technology for vulvovaginal rejuvenation.

Despite the increasing popularity of labiaplasty techniques internationally, little effort has been placed on comparing technique and establishing standardized measurements or guidelines for this procedure. This study, as well as others have been limited by lack of consensus on the definition of labia hypertrophy itself. For this reason, we evaluated labia protrusion as well as hypertrophy as separate entities. Hypertrophy was measured pre and posttreatment by placing the labia on lateral stretch from the vaginal introitus and calculating this distance from lateralmost portion of labia to introitus. Protrusion was measured by determining the distance that the labia minora protrudes beyond the majora (rather than introitus). Further, we obtained data related to patient symptoms which ultimately guided patient selection. There are a number of limitations inherent in the retrospective nature of this study, including the potential for data inaccuracies and confines in study design. The limited number patients and follow-up in this study is the result of pilot data that precede as prospective study currently in place. It may have been beneficial to have a control or sham arm to account for the potential effect of only passing the RF cannula without energy. Despite these limitations, this study represents among the earliest reports of bipolar RF for treatment of labia hypertrophy.

## CONCLUSIONS

Treatment of labia hyperplasia and laxity with bipolar RF may potentially fill a treatment gap of women seeking aesthetic and functional improvements without surgical labiaplasty. A powered prospective randomized double blinded study is needed to further elucidate the role of this technology.
